# The Impacts of Subthalamic Nucleus-Deep Brain Stimulation (STN-DBS) on the Neuropsychiatric Function of Patients with Parkinson's Disease Using Image Features of Magnetic Resonance Imaging under the Artificial Intelligence Algorithms

**DOI:** 10.1155/2021/9915206

**Published:** 2021-07-08

**Authors:** Wei Chen, Maode Wang, Ning Wang, Changwang Du, Xudong Ma, Qi Li

**Affiliations:** Department of Neurosurgery, The First Affiliated Hospital of Xi'an Jiaotong University, Xi'an, Shaanxi Province 710061, China

## Abstract

This study was to explore the effect of subthalamic nucleus- (STN-) deep brain stimulation (DBS) on the neuropsychiatric function of Parkinson's disease (PD) patients using the magnetic resonance imaging (MRI) image analysis technology and the artificial intelligence (AI) algorithm. In this study, 40 PD patients admitted to our hospital from August 2018 to March 2020 were selected as the research objects, and they were divided into a control group and an observation group according to the random number table method, with 20 cases in each group. The patients in the control group were given oral treatment with levodopa tablets; and patients in the observation group were treated with STN-DBS + levodopa tablets. In patients, MRI examinations were performed before and after the treatment, and the image optimization processing algorithm under AI was adopted to process the images. The MRI imaging results of the two groups of patients were observed, analyzed, and compared before and after treatment; and the sports, cognition, and mental states of the two groups of patients were analyzed. It was believed that the MRI image before using the AI algorithm was blurry, and the image was clear after the noise reduction optimization process, which was convenient for observation. The data analysis revealed that the signal-to-noise ratio (SNR) after denoising (32.41) and structural similarity (SSIM) (0.79) had been improved. The results of the study suggested that the space occupation and bleeding symptoms of the two groups of patients were reduced after treatment, and those in the observation group were better than those of the control group; the incidences of dyskinesia and motor symptom fluctuations in the observation group were 5% and 0%, respectively, which were lower than those in the control group (35% and 25%, respectively). After treatment, the Unified Parkinson's Disease Rating Scale (UPDRS) score of the two groups of patients decreased, and it was lower in the observation group than in the control group; and the Montreal Cognitive Assessment (MoCA) and Mini-Mental State Scale (MMSE) scores increased, and those in the observation group were higher in contrast to those in the control group (all *P* < 0.05). STN-DBS was beneficial to improve the clinical symptoms of patients and delay the progress of the disease, and MRI based on AI algorithms can effectively observe the changes in the neuropsychiatric function of patients, which was conducive to further clinical diagnosis and treatment.

## 1. Introduction

Parkinson's disease (PD) is a clinically common neurodegenerative disease, which occurs mostly in the elderly. The average age of onset is about 60 years. PD is less common in young people under 45 years of age [1]. According to research statistics, the prevalence of PD in people over 65 years of age in China is about 1.73% [2]. Most patients with PD are sporadic, and only 6%–10% of patients have family histories.

The main pathological change of PD is the degeneration and death of dopaminergic neurons in the substantia nigra of the midbrain, which causes a significant decrease in the dopamine content in the substantia nigra of the striatum and causes disease [3]. The exact cause of this pathological change is still unclear. Genetic factors, environmental factors, aging, and oxidative stress may be involved in the degeneration and death of dopaminergic neurons in PD. In addition to aging and genetic factors, brain factors such as trauma, smoking, and coffee drinking may also increase or decrease the risk of PD [4, 5]. After the patient is sick, clinical symptoms such as static tremor, bradykinesia, muscle rigidity, and posture and gait disturbance may appear, which seriously affect the quality of life of the patient [6]. At present, the comprehensive treatment methods are adopted in clinics such as drugs, behavioral intervention, and instrumental therapy, which can reduce the clinical symptoms of patients to a certain extent and delay the disease process, but the onset is slow, and the desired result is not reached [7].

The subthalamic nucleus- (STN-) deep brain stimulation (DBS) treatment is to improve the neurological function of the patient through high-frequency electrical stimulation. In previous studies, the efficacy of STN-DBS has been confirmed [8]. Magnetic resonance imaging (MRI) is a frequently used clinical examination method. It can use different pulse sequences to obtain information on human tissues and structures, with the advantages of high resolution, rich contrast, and low radiation [9, 10]. Artificial intelligence (AI) algorithms can perform image reconstruction, tissue segmentation, and other preprocessing for MRI imaging, which are beneficial to improve the clarity and rationality of the final imaging, thereby facilitating the clinical judgment. In this study, the specific therapeutic effects of STN-DBS were analyzed deeply, and the characteristics of MRI images were analyzed based on the AI algorithms, aiming to discuss the impacts of STN-DBS on the neuropsychiatric function of PD patients. The results of the study were reported as follows.

## 2. General Information and Methods

### 2.1. Research Objects

40 PD patients admitted to our hospital from August 2018 to March 2020 were selected as the research objects and divided into a control group and an observation group according to the random number table method, with 20 cases in each group. There was no significant difference in general information such as gender, age, course of the disease, clinical symptom classification, disease classification, comorbidities, and past history between the two groups of patients (*P* > 0.05), but the two groups were comparable (as given in Table 1). The medical ethics committee of our hospital reviewed and approved this study. The diagnostic criteria were determined according to the specification given in Diagnostic Criteria for Parkinson's Disease. The inclusion criteria were defined as follows: patients who met the above diagnostic criteria; patients who had not received other drug treatments in the past 3 months; and those who had informed consent and signed the consent form. The exclusion criteria were determined as follows: those with other malignant tumors; those with secondary PD; those who were allergic to the treatment drugs involved in this study; those with severe cognitive impairment; and those with poor compliance.

### 2.2. Methods

#### 2.2.1. Treatment Schemes

The patients in the control group were given oral treatment with levodopa tablets (manufacturer: Jiangsu Shenlong Pharmaceutical Co., Ltd.; National Medicine Standard: H32024369; specification: 0.25 g) (2–4 times/d, 0.25 g/d, after meals). The patients in the observation group were treated with STN-DBS on the basis of taking levodopa tablets. The specific treatment scheme was as follows. Firstly, the patient was anesthetized locally to install the Leksell stereotactic frame (manufacturer: Swedish Elekta). After the installation was completed, the STN was located using 5.0 T high-resolution MRI. When the bilateral STN was implanted in type 3389 electrodes, subvenous anesthesia was performed to implant an electrical stimulation generator under the clavicle. After 15 days, the voltage, frequency, stimulation location, and pulse width were adjusted with a remote control computer to ensure the best treatment effect and the smallest incidence of adverse reactions. The electrical stimulation was continued for the disease.

#### 2.2.2. MRI Analysis Based on AI Algorithm

MRI examinations were performed before and after treatment. Firstly, before the examination, the research subjects had to be informed about the safety of MRI, including the safety of ferromagnetic materials, the safety of gradient field noise, and the safety of the fetus; and they were informed with the whole process of the examination, including the sequence, function, and examination time of MRI. The appropriate breath-holding training was required for each patient before examination. The patient had to sign informed consent. On the day of the examination, the patient was required to fast for 4–6 hours and empty the bladder before the examination; the patient was required to remain as still as possible during the examination and not to touch the inner wall and wires of the machine to prevent injuries.

The patient was examined with a 5.0 T superconducting magnetic resonance magnetically sensitive weighted imaging scanner (manufacturer: Siemens Avanto; model: Optima 360), using a cranial coil. Sequence scans were performed, including conventional axial T1-weighted images (T1WI sequence) and T2-weighted images (T2WI sequence). The parameters during scanning were set as follows. The parameters for horizontal axis position T1Wl were defined as follows: repetition time (TR): 340 ms, echo time (TE): 2.71 ms; field of view (FOV): 220 mm × 230 mm, matrix: 320 × 320, and layer thickness: 5.0 mm. Parameters for horizontal axis position T2WI were set as follows: TR: 5,000 ms, TE: 76 ms, FOV: 220 mm × 230 mm, matrix: 320 × 320, and layer thickness: 5.5 mm. Parameters for the fluid-attenuated inversion recovery (FLAIR) sequence in the horizontal axis position were given as follows: TR: 8,000 ms, TE: 113 ms, and FOV: 160 × 160 mm. The magnetic resonance used the 3D gradient echo sequence at high resolution, and the imaging parameters were as follows: TR: 30 ms, TE: 21 ms, slice thickness: 1.3 mm, slice interval: 1.5 mm, flip angle: 17°, FOV: 160 mm × 160 mm, matrix: 384 × 220, number of layers: 80 layers, and scan time: 3 minutes and 46 seconds. For some patients, enhanced scan could be performed after the routine scan. The phase map and phase-intensity fusion map could be obtained after required processing.

#### 2.2.3. Image Preprocessing Based on AI Algorithm

The images were processed with the image optimization processing algorithm and denoising algorithm under AI algorithm. The image optimization processing algorithm included image reconstruction, image enhancement, and edge detection. In the image reconstruction process, the filter operator could be optimized to reduce the impacts of projection noise on the reconstruction result, and the high-resolution image could be reconstructed to eliminate and reduce the effect of mixing. During the image reconstruction, a pixel value of the low-resolution image had to be weighted:

(1) $$\begin{array}{c} A_{m,n}=\sum_{s=1}^{P}Q_{m,n,s}X_{s}+l_{m,n}. \end{array}$$

In the above equation, *m* = 1, 2, 3, ...; *n* = 1, 2, 3, ...; *P* = *l*2 m; *A*_*m,n*_ represents the element in the *n*th frame; the weighted value *Q*_*m,n,s*_ represents the contribution of the *s*th high-resolution pixel to the pixel value of the *n*th frame; and *l*_*m,n*_ represents the influence of the additional noise on the pixel value of the *n*th frame.

In image enhancement, it could highlight some information in an image according to specific needs while weakening or removing some unwanted information, during which the following equation was necessary:

(2) $$\begin{array}{c} W\left(g_{i}\right)={\left\|b-jg_{i}\right\|}^{2}. \end{array}$$

In equation (2), *g*_*i*_ represents the inferred restored image represented by individual *I*; *b* refers to the observed degraded image, and *j* is the point spread function in the degradation process.

Image segmentation was used to select the best threshold using the gray features of the image, divide the pixels in the image, and then perform coding and edge detection.

**Figure 1:**
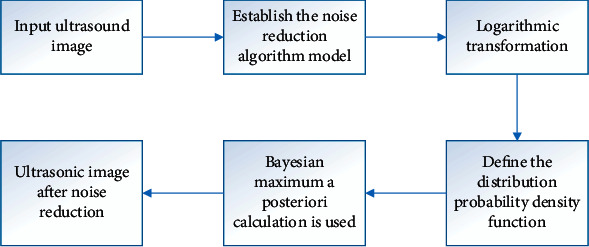
Image processing by the denoising algorithm.

Then, the image was processed using the denoising algorithm (as shown in Figure 1). Firstly, the characteristics of the noise were recorded statistically. The noise approximation model was defined as follows:

(3) $$\begin{array}{c} y=l{}^{*}i+a. \end{array}$$

In equation (3), *l* refers to the noise-free image, *i* represents the multiplicative speckle noise, *a* represents the additive noise, and *y* refers to the corresponding noisy image of *l*. In the ultrasound imaging, the additive noise was very small compared to the multiplicative noise, so *y* could be ignored, and then equation (3) could be written as the following equation:

(4) $$\begin{array}{c} y=l{}^{*}i. \end{array}$$

To separate the noise from the signal, equation (4) could be logarithmically transformed as the following equation:

(5) $$\begin{array}{c} \log\;y=\log\;l+\log\;i, \end{array}$$

or it could be expressed as the following equation:

(6) $$\begin{array}{c} f=m+n. \end{array}$$

In equation (6), *f* = log  *y*, *m* = log  *l*, and *n* = log  *i*. After the statistical characteristics of speckle noise *t* were introduced after logarithmic transformation, the distribution probability density function could be defined as follows:

(7) $$\begin{array}{c} r_{t}\left(t\right)=\frac{t}{d^{2}}\exp-\frac{t^{2}}{2d^{2}},\;t\geq 0. \end{array}$$

In the above equation, *t* represents the amplitude of the noise, and *d* represents the attenuation parameter. Then, the definition of the probability density function of noise was expressed as the following equation:

(8) $$\begin{array}{c} r_{t}\left(t\right)=\frac{t}{d^{2}}\exp-\frac{t^{2}}{2d^{2}}s\left(t\right)-\frac{t}{d^{2}}\exp-\frac{t^{2}}{2d^{2}}s\left(-t\right). \end{array}$$

In the above equation, *s*(*t*) is a step function:

(9) $$\begin{array}{c} s\left(t\right)=\left[\begin{array}{c} 1,\;t\geq 0\\ 0,\;t<0 \end{array}\right]. \end{array}$$

In equation (6), the Bayesian maximum a posteriori calculation method was adopted to calculate noise-free image *z* from the noisy image. The specific calculation equation is given as follows:

(10) $$\begin{array}{c} z\left(y\right)=\arg\max h_{z\left\|\right)y}\left(z\left\|\right)y\right). \end{array}$$

Then, the following equation could be obtained based on the Bayes criterion:

(11) $$\begin{array}{c} z\left(y\right)=\arg\max\left[h_{z\left\|\right)y}\left(y\left\|\right)z\right)*h_{z}\left(z\right)\right]=\arg\max\left[h_{t}\left(y-z\right)*h_{z}\left(z\right)\right], \end{array}$$

which could be transferred into the following equation:

(12) $$\begin{array}{c} z\left(y\right)=\arg\max\left[\log\;h_{t}\left(\left|y-z\right|\right)+\log\;h_{z}\left(z\right)\right]. \end{array}$$

After equations (8) and (9) were incorporated into equation (12), the right side of equation (12) was used to derive *z*, and the result was set to 0, the Bayesian maximum estimation of noise-free image *z* could be obtained with the following equation:

(13) $$\begin{array}{c} z\left(y\right)=y-\frac{d^{2}}{2d^{2}}sign\left(z\right)-\frac{d^{4}}{2d_{z}^{2}}+d^{2^{1/2}}. \end{array}$$

As a result, the image processing by the denoising algorithm could be described in Figure 1.

### 2.3. Observation Indicators

1. The MRI results of the two groups of patients before and after treatment were observed and compared.
2. The exercise, recognition, and mental status of the two groups of patients were evaluated comprehensively based on the results of UPDRS, MoCA, and MMSE. The higher the score in the UPDRS, the more serious the patient's condition; the full score of MoCA was 30, and the score higher than 26 was considered normal; and the lower the score of MMSE, the worse the patient's mental state.
3. The incidences of dyskinesia and motor symptom fluctuations of the two groups of patients were recorded and compared.

### 2.4. Statistical Methods

SPSS 22.0 statistical software was applied for data analysis. The measurement data were represented by $\bar{x}\pm s$, and *t*-test was performed; the count data were represented by case (%), and *χ*^2^ test was performed. *P* < 0.05 indicated that the difference was statistically significant.

## 3. Results

### 3.1. Analysis Results of AI Algorithm

**Figure 2:**
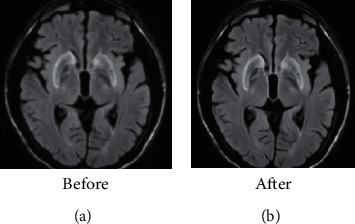
Images before and after processing with the AI algorithm.

Figure 2 shows that the MRI image before using the AI algorithm was blurry, but the image was clear after the noise reduction optimization processing, which was more conducive to observation. The data analysis results proved that the SNR and the SSIM of the image after denoising were greatly improved. The result data of the AI algorithm are shown in Table 2.

### 3.2. Imaging Results of MRI Based on AI Algorithm

**Figure 3:**
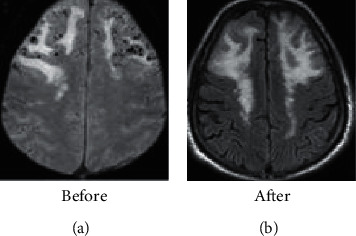
MRI images of patients in the control group.

**Figure 4:**
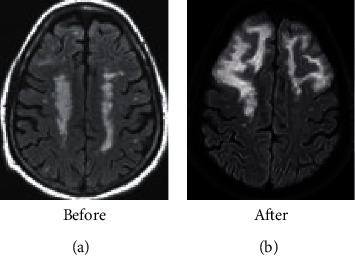
MRI images of patients in the observation group.

Figures 3 and 4 show that, before treatment, both groups of patients showed a slight increase in bilateral frontal white matter edema, a slight increase in the space-occupying effect, a shift of the midline to the left, and a gradient echo sequence. There was bleeding on both sides. After treatment, it was found that the space-occupying effect and bleeding symptoms of the two groups of patients were reduced, and the observation group showed better effect than the control group.

### 3.3. Results of UPDRS, MoCA, and MMSE of Patients in Different Groups

**Figure 5:**
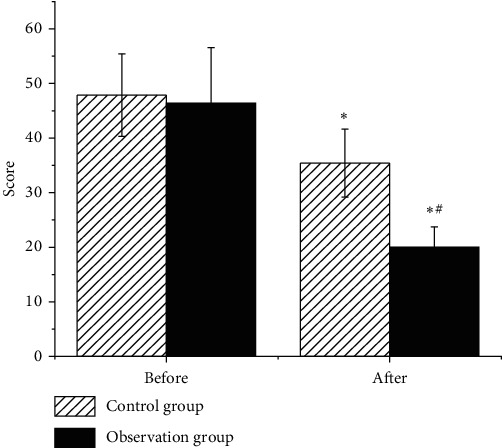
Comparison on UPDRS scores of patients in each group. Note: ^∗^ indicates that the difference was observable in contrast to the score before the treatment (*P* < 0.05); # suggests that there was an obvious difference compared to the control group (*P* < 0.05).

**Figure 6:**
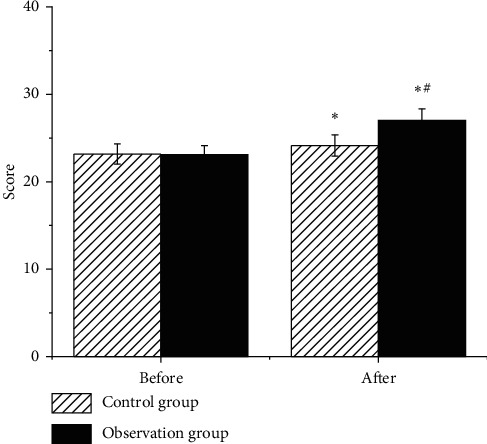
Comparison on MoCA scores of patients in each group. Note: ^∗^ indicates that the difference was observable in contrast to the score before the treatment (*P* < 0.05); # suggests that there was an obvious difference compared to the control group (*P* < 0.05).

**Figure 7:**
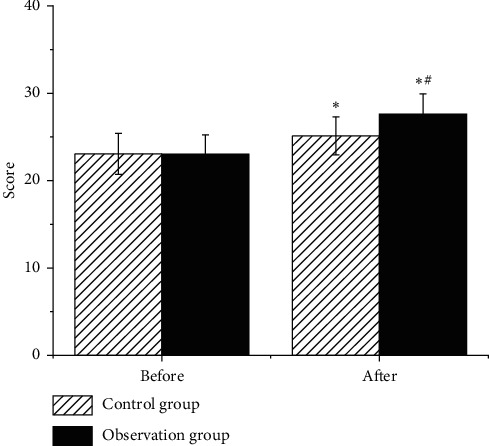
Comparison on MMSE scores of patients in each group. Note: ^∗^ indicates that the difference was observable in contrast to the score before the treatment (*P* < 0.05); # suggests that there was an obvious difference compared to the control group (*P* < 0.05).

As illustrated in Figure 5, the UPDRS scores of the two groups of patients decreased after treatment, and the observation group showed obviously lower score in contrast to the control group. Figures 6 and 7 reveal that the MoCA and MMSE scores of the two groups of patients increased after treatment, and they were higher in the observation group in contrast to those of the control group, showing statistical difference (*P* < 0.05).

### 3.4. Incidences of Dyskinesia and Motor Symptom Fluctuations of Patients in Two Groups

As given in Table 3, the comparison revealed that the incidences of dyskinesia and motor symptom fluctuations in the observation group were greatly lower than the incidences in the control group, showing statistical differences (*P* < 0.05).

## 4. Discussion

Electrical stimulation can change the activity of neurons in the thalamus. STN-DBS is composed of transmission neurons, which mainly send control signals to actions. It is connected to the lateral globus through a part of the basal ganglia, and the internal measurement of the globus can be affected by the afferent signals from the lateral globus pallidus and STN; the STN can also deliver axons to another regulatory area (including the cerebral peduncle-pontine complex) and ultimately achieve the therapeutic effect. Relevant researchers pointed out that the UPDRS score of patients in the postoperative STN-DBS combined state of medication can be greatly improved compared with the preoperative “no medication” state [11–13]. In addition, some researchers also pointed out that STN-DBS can dramatically improve the symptoms of PD patients such as tremor, bradykinesia, and rigidity [14, 15]. The results of this study found that the UPDRS scores of the two groups of patients decreased after treatment, and they were lower in the observation group; and the MoCA and MMSE scores of the two groups of patients increased after the treatment, and they were higher in the observation group. Such results indicated that STN-DBS was beneficial to improve the clinical symptoms of patients and delay the progress of the disease, which was similar to the results of Malek et al. [16].

MRI is a spin magnetic resonance phenomenon, which was discovered on the basis of solid-state microscopic quantum theory and the development of radio microwave electronics technology [17, 18]. Clinical medicine and medical research in front of slavery found that it not only had fast scanning speed but also multisequence imaging, providing more abundant image information for clarifying the nature of the disease. However, it had the disadvantages of uneven magnetic field and large noise, which led to lower imaging clarity. Therefore, it was necessary to use AI algorithms for noise reduction processing [19, 20]. After the AI algorithm was applied to optimize the processing in this study, it was found that the occupation and bleeding symptoms of the two groups of patients were reduced, and the observation group was better than the control group. The scoring of the neurological function and cognitive function of the patients revealed that the MRI examination results were basically consistent with it, suggesting that MRI based on AI algorithms was conducive to accurately observing the changes in the neuropsychological function of PD patients, which was conducive to further clinical diagnosis and treatment.

## 5. Conclusion

In summary, MRI based on the AI algorithm was beneficial to accurately observe that the UPDRS scores of PD patients after STN-DBS treatment were reduced, the MoCA and MMSE scores were both increased, and the incidences of dyskinesia and motor symptom fluctuations were greatly lowered. In addition, MRI examination before treatment showed a mild increase in bilateral frontal white matter edema, slight increase in the space-occupying effect, shift of the midline to the left, and bilateral bleeding in the gradient echo sequence. After treatment, the MRI examination of two groups of patients showed that the space-occupying effect and bleeding symptoms were alleviated. Such comparative results suggested that STN-DBS was beneficial to improve the clinical symptoms and delay the disease progression of patients, and AI algorithm-based MRI could effectively observe the changes in the neuropsychiatric function of patients, which was conductive to further clinical diagnosis and treatment. Therefore, the above treatment and examination methods were worthy of further promotion and use in clinical practice. However, the sample size of the study was small, and the observation indicators in the research process were small, which had specific impacts on the result data. Therefore, the sample size had to be expanded for further research so as to provide more accurate reference data for the clinical diagnosis and treatment.

## Figures and Tables

**Table 1:** Comparison on the basic data of patients in two groups.

	Control	Observation	*P* value
Males	11	13	*P* > 0.05
Females	9	7	
Age (years old)	74.09 ± 2.67	74.33 ± 2.16	
Course of the disease	6.11 ± 2.07	6.46 ± 2.19	
Clinical symptoms			
Stiffness and less exercise	4	5	
Tremor	9	7	
Hybrid	7	8	
Hoehn–Yahr grade			
Grade II	6	5	
Grade III	5	4	
Grade IV	4	6	
Grade V	5	5	
Complications			
Hypertension	12	10	
Diabetes	9	13	
Hyperlipidemia	7	8	
Previous stroke history	13	12	

**Table 2:** Result data of the AI algorithm.

AI denoising algorithm	SNR	SSIM
Before denoising	28.99	0.43
After denoising	32.41	0.79

**Table 3:** Incidences of dyskinesia and motor symptom fluctuations.

	Dyskinesia	Motor symptom fluctuations
Control group	35% (7/20)	25% (5/20)
Observation group	5% (1/20)	0 (0/20)
*P* value	<0.05	<0.05

## Data Availability

The data used to support the findings of this study are available from the corresponding author upon request.
